# Purinergic signaling mediates neuroglial interactions to modulate sighs

**DOI:** 10.1038/s41467-023-40812-x

**Published:** 2023-08-31

**Authors:** Liza J. Severs, Nicholas E. Bush, Lely A. Quina, Skyler Hidalgo-Andrade, Nicholas J. Burgraff, Tatiana Dashevskiy, Andy Y. Shih, Nathan A. Baertsch, Jan-Marino Ramirez

**Affiliations:** 1 Center for Integrative Brain Research, Seattle Children’s Research Institute, Seattle, WA 98101 USA; 2 Department of Physiology and Biophysics, University of Washington, Seattle, WA 98195 USA; 3 Center for Developmental Biology and Regenerative Medicine, Seattle Children’s Research Institute, Seattle, WA 98101 USA; 4 Department of Pediatrics, University of Washington School of Medicine, Seattle, WA 98195 USA; 5 Department of Bioengineering, University of Washington, Seattle, WA 98195 USA; 6 Department of Neurological Surgery, University of Washington School of Medicine, Seattle, WA 98195 USA

**Keywords:** Neurophysiology, Astrocyte, Respiration

## Abstract

Sighs prevent the collapse of alveoli in the lungs, initiate arousal under hypoxic conditions, and are an expression of sadness and relief. Sighs are periodically superimposed on normal breaths, known as eupnea. Implicated in the generation of these rhythmic behaviors is the preBötzinger complex (preBötC). Our experimental evidence suggests that purinergic signaling is necessary to generate spontaneous and hypoxia-induced sighs in a mouse model. Our results demonstrate that driving calcium increases in astrocytes through pharmacological methods robustly increases sigh, but not eupnea, frequency. Calcium imaging of preBötC slices corroborates this finding with an increase in astrocytic calcium upon application of sigh modulators, increasing intracellular calcium through g-protein signaling. Moreover, photo-activation of preBötC astrocytes is sufficient to elicit sigh activity, and this response is blocked with purinergic antagonists. We conclude that sighs are modulated through neuron-glia coupling in the preBötC network, where the distinct modulatory responses of neurons and glia allow for both rhythms to be independently regulated.

## Abstract (web summary)

Sighs are augmented breaths necessary to maintain normal breathing. Here, the authors show that sighs are generated within the preBötzinger complex by emergent network properties that involve neuroglial interactions mediated by purinergic signaling as well as intrinsic and extrinsic modulatory inputs.

## Introduction

Rhythmic activity is ubiquitous and dictates life from birth to death. Cortical activity states in wakefulness and sleep are characterized by multiple frequencies and rhythmic properties^1,2^. Among the most important rhythms of life, breathing controls not only ventilation and vocalization but also serves as an important timing mechanism for cortical activity^2–7^. Under normal conditions, inspiratory efforts occur in two forms, eupnea and sighs. Eupnea refers to normal, resting breathing, which is periodically interrupted by deep, large-volume inspirations called sighs. Eupnea maintains blood gas homeostasis, whereas sighs function to prevent the spontaneous collapse of alveoli that occurs over time, a process termed atelectasis^8^. Moreover, sighs seem to precede most awakenings, and their frequency increases during sleep transitions, when exposed to hypoxia, and during emotions of relief, concentration, and love^8–10^.

Inspiration is generated and shaped by a spatially dynamic network located bilaterally along the ventrolateral medulla^11^. Within this network, the pre-Bötzinger Complex (preBötC) constitutes a core that is both necessary and sufficient for inspiration^12–16^. The preBötC is heterogenous, containing primarily glutamatergic, GABAergic, and glycinergic neurons. Many preBötC neurons also express sodium- and Ca^2+^-dependent bursting currents (I_NaP_ and I_CAN_, respectively), and those that express a high density of either of these currents can become autorhythmic, (i.e., have the ability to fire bursts of action potentials in the absence of synaptic inputs)^17–20^. Although glutamatergic synaptic interactions in the preBötC may be the minimum requirement for producing an inspiratory rhythm^21^, a dynamic eupneic rhythm emerges from heterogeneous interactions among synaptic excitation, inhibition, and intrinsic bursting currents^22^. Indeed, mechanisms that underlie eupnea generation in the preBötC have been an active area of research for nearly two decades^12,22–28^.

Much less is known about the mechanisms underlying sigh generation. The ability to record directly from the preBötC in thin brainstem slices has revealed that this network can simultaneously generate two distinct rhythms—a fast eupneic rhythm and a slow, larger amplitude, sigh rhythm^29^. Indeed, even when further isolated in “micro islands”, the preBötC continues to produce eupnea and sighs^30^. Since these activities are described in reduced, in vitro preparations, they are often referred to as “fictive eupnea” and “fictive sighing”; however, the characteristics of eupnea and sigh activities generated in vitro and in vivo are strikingly similar^31^. Intracellular patch recordings from the isolated preBötC revealed that many neurons are active during both eupnea and sighs^30^ and that some autorhythmic preBötC neurons continue to generate sigh-like bursts even when synaptic interactions are blocked, suggesting that these rhythms are not generated by distinct neuronal circuits. Yet, the spatial extent of the active inspiratory network expands during sighs^32^, and a small population of “sigh-only” neurons has been identified^30^, suggesting that the preBötC recruits additional neurons to participate in the inspiratory rhythm during sighs. Thus, the basic elements required for the generation of eupnea and sigh activity are largely overlapping and are contained within the preBötC.

Despite being generated by the same neuronal circuit, eupnea and sigh activities can be differentially modulated. These modulatory differences may be physiologically important during hypoxia or sleep/wake transitions when sighs are specifically upregulated^8,33–36^. Some of these modulatory influences likely originate from regions located outside the preBötC^37,38^. This includes the more rostrally located retrotrapezoid/parafacial respiratory group (RTN/pFRG), which may modulate sighs via peptidergic projections to the preBötC^39^; however, sighs are not eliminated by lesions of this region, blurring the role of the RTN/pFRG^40^. The nucleus tractus solitarius (NTS), an important region mediating respiratory responses to hypoxia, is also likely able to modulate sighing. Within the preBötC, blocking high-threshold voltage-gated P/Q type Ca^2+^ channels abolishes sighs, but not eupneic activity^41^. Eupnea and sigh rhythms are also differentially modulated by metabotropic glutamate receptors^42^ and a variety of aminergic and peptidergic neuromodulators^30,31,39,43–45^. These neuromodulators act through G-protein coupled receptors that activate downstream pathways to increase intracellular Ca^2+^, consequently reshaping Ca^2+^ dynamics in the network^46^, suggesting that sigh generation in the preBötC may involve the regulation of Ca^2+^ dynamics.

Glial cells play important roles in modulating the breathing rhythm^47^, and like neurons, glial activity is regulated by neuromodulators^48^. Glia mediate chemosensitive and pH-dependent breathing responses by propagating ATP-induced Ca^2+^ excitation^49–54^. Moreover, glia located near the PreBӧtC express small membrane currents that are purportedly phase-locked with respiratory activity^54^. Because Ca^2+^ fluctuations in glia have been broadly implicated in neuronal network function^55,56^, including the control of network synchronization by up and down-regulating synaptic transmission^57,58^, we hypothesized that Ca^2+^ dynamics within glial cells are a key mechanism that allows the preBötC network to simultaneously generate eupnea and sigh rhythms.

To address the question of how sighs and eupnea are generated in the same network, we combined in vitro slice electrophysiology, pharmacology, imaging techniques, and in vitro and in vivo optogenetics to explore how neurons and glia interact to generate sighs. Using pharmacology in a live slice preparation of the preBötC, we recorded neural population activity of eupnea and sigh rhythms. Our results revealed that purinergic P2Y1-dependent mechanisms are required for sigh generation and that P2Y_1_ receptor (P2Y_1_R) inhibition blunts the augmented portion of the hypoxic response by blocking sighs. Furthermore, we report that the sigh rhythm is not dependent on inhibitory interactions, as blockade of inhibition does not alter sigh characteristics or frequency. Targeted optogenetic stimulation of preBötC astrocytes was sufficient to elicit sigh and eupnea activity, which was blocked by purinergic P2Y_1_R antagonists. Multiphoton imaging in preBötC slices further revealed that β-adrenergic receptor (βA_R_) activation increases intracellular Ca^2+^ activity, corresponding to an increase in sighs. Based on these findings, we conclude that sighs are an emergent property of the preBötC network, regulated by neuroglial interactions to modulate sigh rhythmogenesis.

## Results

### Characterization of sighs: sighs are not generated through inhibitory mechanisms

**Fig. 1:**
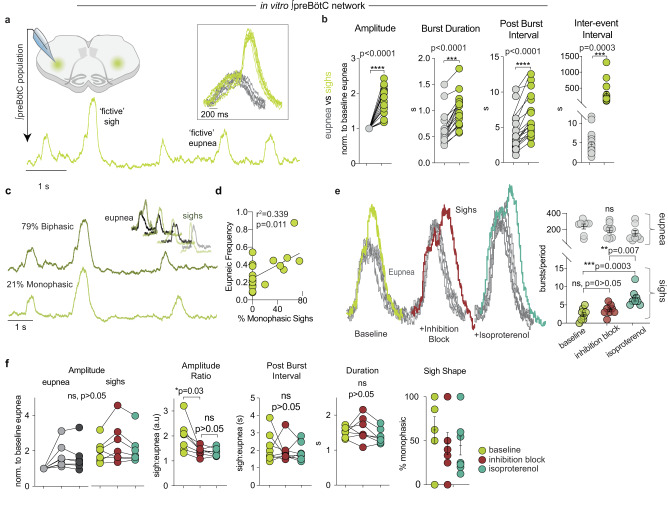
In vitro comparison of eupnea and sigh characteristics. **a** Integrated population activity of eupnea and sighs recorded from the transverse slice preparation. Inset shows overlaid ‘fictive’ eupnea (gray) and sighs (green) for comparison. **b** Amplitude, burst duration, post-burst interval, and inter-event interval of eupnea and sighs differ significantly (one-tailed paired *t*-test, *n* = 18,). **c** Examples of monophasic and biphasic sigh shapes and comparison to eupnea. **d** Monophasic sighs increase with increasing eupneic frequency (linear regression). **e** Inhibition block (strychnine and gabazine) does not alter sigh frequency at baseline or with βA_R_ agonist isoproterenol (*n* = 8, ordinary one-way ANOVA) and **f** does not alter sigh amplitude, post-burst interval, duration, or shape (*n* = 7, ordinary one-way ANOVA). Inhibition block does result in an increase in eupnea amplitude, decreasing the sigh:eupnea ratio (ordinary one-way ANOVA). Data are presented as mean values ± SEM.

The neuronally recorded in vitro preBötC population activity is referred to as ‘fictive’ breathing or sighing, and integrated population burst activity is referred to as a burst. Fictive sighs in vitro and sighs in vivo are characterized by their large amplitude, extended burst duration, the period of time following the occurrence of a sigh and onset of the next eupneic breath, and temporal occurrence. While sighs vary in shape, not every large amplitude burst is defined as a sigh. For example, sighs can occur with a similar amplitude to eupnea depending on network stability^59^, but retain an extended duration and post-burst interval. A failure to appropriately identify sighs can lead to considerable misinterpretation of data. Thus, we analyzed characteristics that distinguish sighs from eupneic bursts generated by the preBötC in brainstem slices from neonatal mice. Under control conditions, sigh amplitude, burst duration, and post-burst interval were significantly greater than eupneic bursts and occurred on a much slower time scale (Fig. 1a, b). Sighs varied in shape depending on their relationship with the eupneic rhythm; some sighs appeared as connected double bursts, with a large amplitude burst superimposed on a eupneic burst (‘biphasic’), whereas others occurred as a single large amplitude burst (‘monophasic’) (Fig. 1c, Supplementary Fig. 1a). Biphasic and monophasic sighs did not differ in burst amplitude, burst duration, or post burst interval. Under control conditions, we found that 78.5 ± 5.8% of total sighs were biphasic, and 21.4 ± 5.8% were monophasic. The prevalence of each sigh shape was variable between slices, and faster eupneic rhythms tended to have more monophasic sighs (Fig. 1d). Pharmacological blockade of GABAergic and glycinergic synaptic inhibition with strychnine and gabazine, referred to as “inhibition block”, did not eliminate sighs, change their frequency, or dissociate them from eupneic bursts (Fig. 1e, f)^60^. Sigh amplitude, post-burst interval, duration, and shape were also unaffected by the inhibition block. However, the amplitude of eupneic bursts increased relative to sighs^32^, thereby decreasing the ratio of sigh to eupnea amplitude (Fig. 1f). Moreover, sigh frequency could still be increased pharmacologically following inhibition block with the βA_R_ agonist isoproterenol (Fig. 1e, f), a potent sigh modulator^31^. Thus, we conclude that inhibitory synaptic interactions among preBötC neurons are not required for sigh generation.

### Astrocytes modulate sighing through purinergic signaling

Glia have recently been found to modulate respiratory rhythmogenesis through several mechanisms^47,51,61–63^. For example, pH-sensitive astrocytes on the ventral surface of the medulla can alter respiratory frequency^51^. These astrocytes release modulatory cues, such as D-serine^63^ and prostaglandin E2^62^, from various brainstem regions, to stimulate the neural network and alter breathing. Astrocytes also respond to metabolic changes in their extracellular environment by increasing intracellular Ca^2+^^50^ through G-protein coupled receptor (GPCR) cascades. GPCR pathways are activated by Norepinephrine (NE), a biogenic amine known to modulate eupnea and sigh behavior^64^. Astrocytes in the medulla stabilize respiratory activity and have been shown to respond to NE with increases in Ca^2+^^48^. Moreover, recent evidence demonstrates that NE is important for astrocyte-induced activity in the cortex during sleep^65^. Thus, we hypothesized that preBötC glia could play a critical role in generating sighing. We first confirmed previous findings that NE modulates sigh activity;^66^ our results confirm that NE increases the frequency of sighs and found that high NE concentrations abolish sighs, but not eupnea activity (Supplementary Fig. 2a–c). NE also increased the proportion of monophasic sighs, coinciding with an increase in the eupneic frequency.

**Fig. 2:**
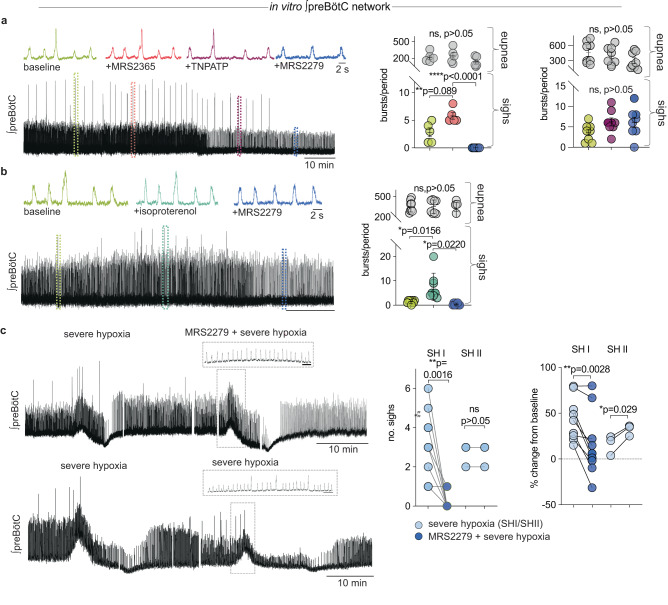
P2Y_1_R-dependent signaling is necessary for spontaneous and hypoxia-induced sigh generation in the preBötC. **a** Integrated preBötC activity depicting time course for P2Y_1_R manipulation. MRS2365 significantly increased the number of sighs from baseline, while P2Y_1_R antagonist MRS2279 abolished all sighs (*n* = 5, one-way ANOVA). P2XR antagonist (TNP-ATP) did not significantly affect sigh frequency (*n* = 8, one-way ANOVA); no significant effect on eupneic frequency was observed with any drug additions. Analysis of sigh and eupnea burst number is reported as number of bursts/20 min period (one-way ANOVA). **b** P2Y_1_R antagonist MRS2279 eliminated the βA_R_ agonist induced increase in sighs (*n* = 8, one-way ANOVA). **c** Representative traces from transverse preBötC slices during the initial bout of severe hypoxia (SH, 6 mins), followed by a recovery period and second hypoxic challenge with MRS2279 (*n* = 9). The lower panel depicts control with two consecutive bouts of severe hypoxic challenge. Gaps in recordings were made to align control and experimental traces. Inset preBötC integrated traces have time scales of 10 s. MRS2279, in addition to severe hypoxia, significantly impaired the hypoxic response by eliminating sighs (left) and reducing the augmenting phase of the eupneic rhythm (right, both two-tailed paired *t*-tests). Data are presented as mean values ± SEM.

In the respiratory network^67,68^ and throughout the central nervous system^69,70^, astrocytic Ca^2+^ signaling involves the release of ATP, which modulates neural activity via purinergic receptors^71–73^. Therefore, we tested how purinergic signaling contributes to sigh generation in vitro by pharmacological manipulation of the ADP and ATP receptors P2Y1 and P2X, respectively (Fig. 2a–c)^74^. Activation of purinergic P2Y_1_Rs with MRS2365 significantly increased sigh frequency (Fig. 2a). Subsequent blockade of P2Y_1_R (MRS2279), but not P2X receptors (TNP-ATP), abolished sighs. The P2Y_1_R antagonist also blocked sighs induced with the βA_R_ agonist isoproterenol (Fig. 2b).

During the onset of hypoxia, eupneic activity increases along with sighing, followed by a transformation of the normal inspiratory rhythm into gasping^30^. To test if purinergic P2Y_1_R inhibition would also block sighs generated during the central hypoxic response in vitro (Fig. 2c)^30,68,71^, eupnea and sigh activity was recorded before and during a bout of severe hypoxia (SH). We applied one bout of severe hypoxia followed by a recovery period before applying MRS2279 during a second hypoxic episode. In addition to the expected suppression of the augmented eupneic frequency response^71^, P2Y_1_R inhibition completely eliminated hypoxia-induced sighs in 8/9 slices. In control experiments where no drugs were applied, there was no change observed in eupnea or sighs during the second exposure to severe hypoxia (*n* = 3, Fig. 2c).

P2Y_1_Rs are expressed by brainstem astrocytes^71,73,75^. We confirmed this finding using standard immunohistochemical staining and observed that P2Y_1_R puncta coincide with neuron (βIII Tubulin) and astrocyte (Aldh1l1) nuclei (Supplementary Fig. 2d).

**Fig. 3:**
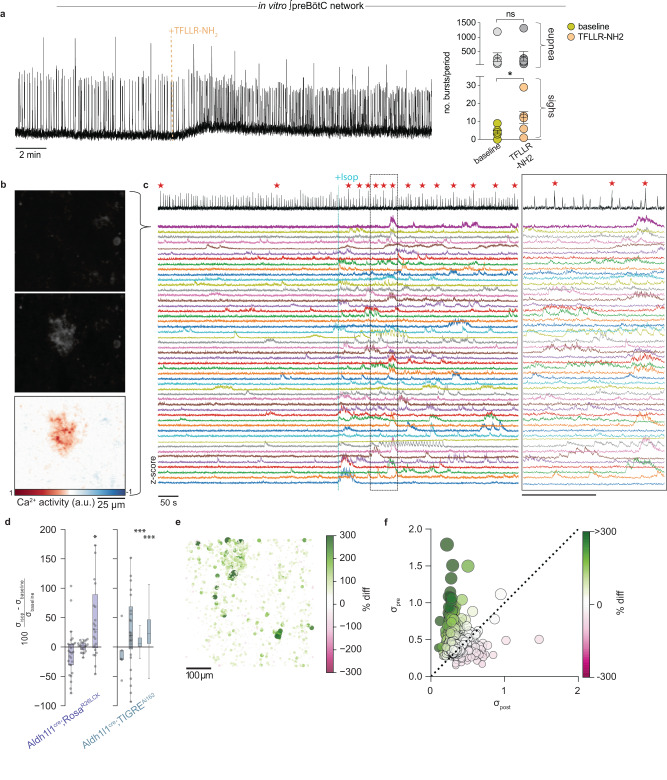
Isoproterenol concomitantly increases sighs and astrocytic Ca^2+^ activity. **a** Par-1 agonist TFLLR-NH2 robustly increased the frequency of sighs (*n* = 8, paired *t*-test, p = 0.01), but did not alter the eupneic frequency (*p* = 0.18). **b** Example ROI of intracellular Ca^2+^ fluorescence before (top) and after (middle) bath application of isoproterenol. Bottom panel shows difference in fluorescence with the addition of isoproterenol. **c** Integrated preBötC activity (top, black) and Ca^2+^ transients from 50 example ROI’s (below, colors) in a representative preBötC slice before and after isoproterenol. Top thick magenta ROI trace is the ROI shown in (**b**). **d** Percent difference in Ca^2+^ activity per ROI after bath application of isoproterenol, for each recorded slice (from left to right, total no ROIs Aldh1l1^cre^;Rosa^R26LCK^ = 27, 19, 23; Aldh1l1^cre^;TIGRE^Ai162^ = 5, 28, 2571, 2520). Color indicates driver/reporter line. Dots are ROIs, horizontal line is median, box is median ± IQR, whiskers are +/1.5x IQR. Two rightmost ALDH1l1^cre^;TIGRE^AI162^ slices had >2000 ROIs, so we omit individual ROI points for visual clarity **e** Heatmap depicting percent difference in Ca^2+^ activity from baseline to isoproterenol for all ROIs in an example slice, mapped to their location on the slice (dorsal is up, medial is right). Larger changes in Ca^2+^ activity are represented by size of circle and dark green color **f** Standard deviation of Ca^2+^ activity for each ROI before (*x*-axis) and after (*y*-axis) isoproterenol application. ROIs that lie above the unity line indicated increases in Ca^2+^ after isoproterenol application. Marker size indicates the standard deviation of ROI Ca^2+^ post-isoproterenol application; marker color indicates the % difference of standard deviation as in (**e**). Data are presented as mean values ± SEM.

Activation of the βA_R_ by the agonist isoproterenol robustly stimulates sighs^31^ without activating eupnea, as does NE. Furthermore, activation of βA_R_s leads to intracellular increases in Ca^2+^ through G-protein signaling and cAMP-mediated PKA activation^64^. Thus, we hypothesized that intracellular Ca^2+^ increases in astrocytes stimulate the release of ATP to drive purinergic signaling, stimulating preBötC neurons to generate sighs. Therefore, we tested pharmacological activation of Ca^2+^ signaling in astrocytes using the peptide TFLLR-NH2 in slices of the preBötC (Fig. 3a). This drug triggers intracellular Ca^2+^ elevations by activation of the PAR-1 receptor (thrombin protease-activated receptor-1), which is highly expressed by astrocytes^76–79^. Our results revealed an increase in sigh frequency with no change in eupnea upon application of 5 μM TFLLR-NH2 (*n* = 7), indicating that increases in intracellular Ca^2+^ in astrocytes could facilitate the observed increase in sigh frequency (Fig. 3a).

### Isoproterenol increases preBötC astrocyte calcium activity

Next, we asked if transmission of Ca^2+^ activity among preBötC astrocytes coincides with sigh generation. We performed 2-photon imaging of astrocytes in preBötC slices using transgenic mouse lines *Aldh1l1*^Cre^;*Rosa26*^*LCK*GCaMP6s^ *and Aldh1l1*^Cre^;*TIGRE*^*Ai162*^ (Fig. 3, Supplementary Fig. 3). All strains exhibited similar responses to isoproterenol. *Aldh1l1*^Cre^;*Rosa26*^*LCK*GCaMP6s^ slices had small, cytoplasmic localized Ca^2+^ activity (referred to as Ca^2+^ ‘sparks’), while *Aldh1l1*^Cre^;*TIGRE*^*Ai162*^ astrocyte activity was observed in soma as well as in processes. Astrocytes within the preBötC and surrounding areas frequently exhibited rhythmic Ca^2+^ transients (Fig. 3c, Supplementary Fig. 3a–f). We observed variations of Ca^2+^ fluctuations in astrocytic end feet surrounding blood vessels, small Ca^2+^ sparks, whole cell Ca^2+^ bursts, and Ca^2+^ waves that extended across multiple cells.

When compared to baseline, isoproterenol increased astrocytic Ca^2+^ activity in 3 of 7 slices (mean 25% increase in ROI activity) as measured by the percent difference in the standard deviation of the Ca^2+^ signal (Fig. 3b–d). During baseline two-photon recordings, 13% (±8.6% S.D) of astrocytic ROIs per slice (*n*_slices_=7) exhibited periodic Ca^2+^ activity (Supplementary Fig. 3f). Furthermore, the percentage of rhythmic ROIs did not significantly change in the presence of 20 μM isoproterenol (15 ± 7% S.D, *p* = 0.55 paired *t*-test, Supplementary Fig. 3f).

To determine if the periodicity of astrocytic Ca^2+^ activity is temporally related to sighs, we computed the sigh-triggered average for each ROI obtained from two slices. We performed a paired *t*-test between the mean Ca^2+^ signal in a window before and after sigh onset, using window sizes of 1, 2, 5, and 10 s. *P*-values for all tested window sizes returned uniform distributions, indicating there is no strict temporal correlation between astrocyte Ca^2+^ activity and sighs (Supplementary Fig. 3e). After Bonferroni correction, only a singular ROI exhibited statistically significant changes in activity relative to sigh onset. Based on the rarity of a strict phasic association between astrocytic Ca^2+^ transients and the occurrence of sigh rhythmic activity, we hypothesize that sigh activity is an emergent property involving neuroglia interactions which is not dictated by a consistent phase-specific activation of sigh glia or neurons.

### Activation of preBötC astrocytes drives sighing behavior

**Fig. 4:**
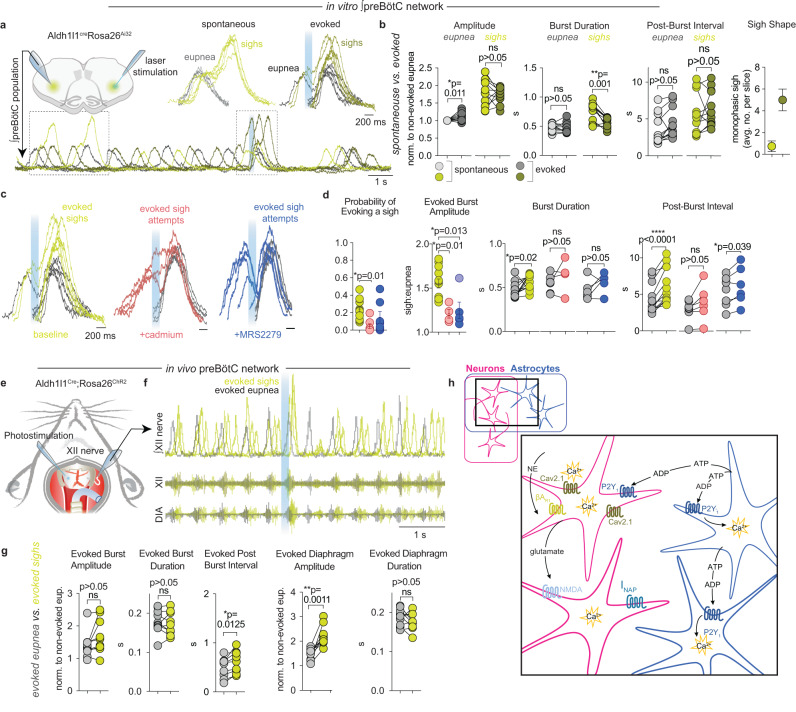
Chr2 activation of preBӧtC astrocytes drives sighing in vitro and in vivo. **a** PreBötC slice depicting electrode and laser placement and representative traces comparing spontaneous (light green, gray) and photo-evoked (dark green, dark gray) sighs and eupnea, with the expanded view above. The turquoise bar represents 200 ms light pulse. **b** Evoked sighs withheld the same characteristics as spontaneous sighs with a smaller duration, given the larger number of evoked monophasic sighs (paired *t*-test, *n* = 12). **c** Optogenetically evoked sighs are blocked by Cadmium (4 µM) and MRS2279 (20 µM). Representative traces depicting differences between baseline evoked sighs and ‘sigh attempts’ and eupnea in drug conditions. **d** Cadmium, but not MRS2279, significantly reduced the probability of evoking a sigh. Sigh characteristics were absent from evoked bursts in cadmium and MRS2279, including a reduction in burst amplitude, burst duration, and post-burst interval (paired *t*-test, *n* = 12). Graphs show that ‘sigh attempts’ did not significantly differ from eupnea in most characteristics. Missing symbols in Cadmium or MRS2279 represent that no sigh attempts were evoked. **e** Schematic depicting in vivo anesthetized preparation and **f** corresponding (shortened) evoked sweeps as recorded from the hypoglossal (XII) nerve. **g** Evoked sighs in vivo were best distinguished by diaphragm amplitude and generally retained sigh characteristics that were significantly different from evoked eupnea, apart from burst duration and post-burst interval (*n* = 11). **h** Hypothesis of sigh rhythm generation. Changes in PO_2_ levels increase intracellular Ca^2+^ in astrocytes, resulting in ATP release, which is converted to ADP. ADP binds to purinergic P2Y_1_Rs leading to a Ca^2+^ increase in astrocytes and neurons through g-protein signaling cascades. P2Y_1_R-driven sighs in vivo can be overridden by neuromodulation from upstream pathways (e.g., Norepinephrine, neuromedin B, gastrin-releasing peptide) to provide further sigh modulation depending on metabolic state. Data are presented as mean values ± SEM.

To further investigate the potential role of astrocytes in sigh generation, we tested whether selective manipulation of astrocytes^80,81^ would be sufficient to affect eupnea and sigh activity. In brainstem preBötC slices from *Aldh1l1*^Cre^;*Rosa26*^ChR2:EYFP^ mice, we found that brief (200 ms, 0.5 mW/mm^2^) light pulses reliably evoked both eupnea and sigh bursts recorded from the contralateral preBötC in 12/25 slice preparations (Fig. 4a, b). In the remaining slices, longer duration or higher intensity light pulses were effective in evoking eupnea and sigh bursts. Sighs evoked by photoactivation of *Aldh1l1*-expressing cells were indistinct from spontaneous sighs in burst amplitude and post-burst interval (Fig. 4a). However, the duration of evoked sigh bursts was shorter relative to spontaneous sighs, which could be explained by the increased number of monophasic sighs (Fig. 4b). These experiments suggest that *Aldh1l1* astrocytes are sufficient to initiate both sigh and eupneic bursts that are largely indistinguishable from spontaneously occurring activities.

Blocking P/Q-type Ca^2+^ channels inhibits sighs^42^, and P/Q type (Ca_V_2.1) knockout mice do not generate sighs in vitro or in vivo and ultimately die of atelectasis^41^. A similar selective inhibition of spontaneous sighing is observed following the administration of low concentrations of the general Ca^2+^ channel blocker Cd^2+^ ^30,82^. Therefore, 4 μM Cd^2+^ was applied prior to optogenetic stimulations to test how suppression of Ca^2+^ channel activity would affect the ability of astrocytes to induce sighs (Fig. 4c, d, Supplementary Fig. 4a). In Cd^2+^, only 4.7% of stimulations (compared to 24.1% at baseline) evoked a sigh-like burst, and those that did occur had characteristics that differed from sighs evoked under control conditions. The amplitude of these “sigh attempts” in Cd^2+^ was significantly reduced relative to control sighs but remained slightly larger than evoked eupnea. The duration of sigh attempts in Cd^2+^ was statistically indistinct from eupnea duration. Moreover, sigh attempts in Cd^2+^ lacked an extended post-burst interval (“post-sigh apnea”) that is characteristic of normal sighs (Fig. 4d).

We also tested if the P2Y_1_R antagonist MRS2279 would affect the ability of astrocytes to drive sighing (Fig. 4c, d, Supplementary Fig. 4a). Following P2Y_1_R inhibition, we observed sigh attempts similar to what we observed in Cd^2+^. Consistent with evoked sigh attempts observed in Cd^2+^, sigh attempts in MRS2279 were smaller in amplitude than baseline sighs and had burst durations similar to evoked eupnea. However, post-burst intervals after sigh attempts in MRS2279 remained longer than those after eupnea (Fig. 4d). Collectively, these data indicate that P2Y_1_R- and Ca^2+^-dependent signaling are important mechanisms that allow astrocytes to drive sighing activity in the preBӧtC.

Next, we asked if light activation of astrocytes in vivo would have the same effect on sigh modulation (*n* = 9). Indeed, we found that there was a higher probability of evoking sighs in vivo than in vitro (Fig. 4d, Supplementary Fig. 4d), and in general, sigh characteristics similarly matched in vitro data. Evoked sighs in vivo could be unambiguously distinguished from eupnea by characterizing the diaphragm amplitude (Fig. 4g). Notably, evoked eupnea in simultaneous recordings of the hypoglossal nerve (XII) tended to be larger in amplitude than spontaneous eupnea, which made distinguishing sighs from eupnea more difficult based solely on XII recordings (Supplementary Fig. 4d).

Interestingly, MRS2279 bilaterally injected into the preBötC in vivo was less effective in completely blocking spontaneous or evoked sighs (*n* = 3/6, Supplementary Fig. 4c, d), contrary to the very reliable inhibitory effect observed in vitro (Fig. 4b). This may not be surprising given that numerous descending inputs that are present in vivo and endogenously modulate sighs^83^ including bombesin-like peptides^39^ and noradrenergic inputs^31^ will likely reduce the ability to block sighs by purinergic mechanisms in vivo. Corresponding to this variability, we observed a higher probability of evoked burst failures altogether in the presence of MRS2279 (Supplementary Fig. 4d). This weaker stimulation corroborates the control of sighs from outside areas^39^, which are eliminated from in vitro slice experiments. Following in vivo experiments, animals were perfused, fixed, embedded and imaged to confirm the location of injection sites was specific to the preBötC (Supplementary Fig. 4e). To verify Aldh1l1^cre^ transgenic lines were specific to astrocytes, we immunostained preBötC slices with neuronal stain NeuN. No crossover between Aldh1l1^cre^/Rosa26^eYFP^ and NeuN was observed (Supplementary Fig. 4f). To further verify the specificity of Aldh1l1 astrocytic marker, we confirmed specificity using fluorescence in site hybridization (FISH) and observed no overlap between Aldh1l1 and NeuN probes (Supplementary Fig. 4g).

Our data indicate that sighs are an emergent property involving local neuroglial interactions mediated by purinergic signaling and modulatory inputs intrinsic and extrinsic to the preBötC.

## Discussion

An explanation for how the preBӧtC generates two temporally distinct rhythms has proven elusive. Studies attempting to unravel the mechanisms of sigh generation have proposed the existence of two neuronal subnetworks within the preBötC^84^, suggesting that a small (5%) population of sigh-only neurons^30,85^ may generate the sigh rhythm based on slow intracellular Ca^2+^ oscillations. However, these sigh-specific neurons do not continue to burst after blocking glutamatergic and glycinergic transmission^30^. Moreover, the published computational model^84^ requires inhibitory synaptic input to generate the characteristic biphasic sigh bursts, whereas our data shows that inhibition is not required for sigh generation. This concept is consistent with recordings from embryonic brainstem^84^ and P0 neonatal^29^ slice preparations^85^; however, our results suggest that neither the biphasic sigh shape nor sigh generation is dependent on inhibitory interactions.

Similar molecular cascades and neuroglial interactions may generally affect respiratory rhythm generation^51,54,62,86–88^ while specifically determining the sigh periodicity. Such mutual interactions could explain the differential roles of neurotransmission and glial modulation of sighs versus normal respiratory activity. For example, different distributions of metabotropic glutamate receptors on glia^89^ and neurons could explain the strikingly different metabotropic responses observed for eupnea and sighs^42^. In the case of sigh-specific β-adrenergic modulation^31^, glia and neurons may possess the relevant modulatory receptors. Indeed, our experiments show that the purinergic P2Y_1_R antagonist completely blocked both spontaneously occurring and neuromodulator-evoked sighs with the β-adrenergic agonist isoproterenol (Fig. 2b). Significantly, we found that while P2Y_1_R agonists and antagonists strongly modulated the sigh, they did not significantly alter the eupneic frequency (Fig. 2a). Increases in intracellular Ca^2+^ in preBötC astrocytes driven by hypoxia results in release of ATP, concurrent activation of ATP binding to P2Y_1_R on neighboring astrocytes, and simultaneous binding of ATP to neuron-specific P2Y_1_Rs to stimulate neuronal activity^71,74,90^ and consequently could result in increased neuronal recruitment to generate a sigh burst. Our Immunostaining is suggestive of glial and neuronal expression of P2Y_1_Rs in the preBötC (Supplementary Fig. 2d). Thus, further exploration of the specific mechanisms that result in bi-directional neuro- and glial activation during the sigh needs to be explored.

We found that sigh frequency was strongly dependent on P2Y_1_Rs, as the P2Y_1_R antagonist strongly blocked the occurrence of sighs. Interestingly, the application of the P2Y_1_R agonist increased the frequency of sighs but was not as effective as β-adrenergic modulation and showed more slice-to-slice variability. Furthermore, the P2Y_1_R antagonist was still able to block the β-adrenergic increase in sighs (Fig. 2b). Neither the purinergic agonist nor antagonist had any significant effect on the eupneic rhythm, which is interesting given that others have reported an increase in respiratory frequency with P2Y_1_R modulation^74,90^. These modulatory effects suggest that sigh emergence in the preBötC is governed by interactions between P2Y_1_Rs on astrocytes that drive the recruitment of neurons expressing β-adrenergic receptors or other receptors that strongly modulate sigh frequency (e.g., oxotremorine, substance P). Furthermore, a mechanism that supports sigh rhythmogenesis through neuroglial interactions in the preBӧtC follows previously identified purinergic mechanisms in both the respiratory networks^67,91–94^ and in other brain areas that coordinate the hypoxic response, such as the carotid bodies^95^. Given that an important physiological role of sighs is to maintain adequate available lung capacity and act as an arousal mechanism in response to hypoxia^8^, a role for O_2_ and CO_2_-sensitive glia in coordinating this activity is plausible. Purinergic activation plays a critical role in potentiating inspiratory synaptic inputs, glutamatergic currents, and persistent inward currents in XII motoneurons. Consequently, a loss of purinergic modulation may contribute to state-dependent reductions in XII motoneuron excitability^96^. It is also worth noting that sighs are typically activated during transitions into sleep^97^, which may also be associated with changes in O_2_, and, consequently, purinergic signaling. Sighs are very sensitive to changes in central PO_2_, as the preBötC responds reliably with the generation of sighs during hypoxic episodes^29,98^. The link with astrocytes, as proposed in the present study, provides a possible explanation for the underlying cellular sequence. Physiological decreases in PO_2_ inhibit glial mitochondrial respiration, which in turn causes mitochondrial depolarization, activation of phospholipase C, IP_3_ receptors and release of Ca^2+^ from intracellular stores, which then triggers the fusion of vesicular compartments and the release of ATP^50^. According to the data presented here, this cascade could activate sighs. As we have shown, the blockade of P2Y_1_R inhibits transmission to the respiratory network and the cessation of sighs.

Since astrocytes in various metabolic states respond by increasing intracellular Ca^2+^^50^, and our preliminary model of the preBötC suggested sighs could be a result of slow Ca^2+^ oscillations, we hypothesized that sighing was dependent on the presence of a slow, sigh-linked calcium signal. Indeed, our preliminary experiments with the PAR-1 receptor agonist TFFLR-NH2 triggered a robust increase in sighs in slices of the preBötC (Fig. 3). While this drug purportedly triggers intracellular Ca^2+^ elevations through activation of PAR-1, expressed by astrocytes^76–79^, we followed these experiments by performing in vitro 2-photon Ca^2+^ imaging. While our results did not reveal a coordinated astrocytic Ca^2+^ oscillation time-locked with sighs, we found that β-adrenergic modulation of preBötC slices leads to an overall increase in Ca^2+^ transients. Interestingly, astrocytes that were responsive to β-adrenergic modulation did not have any clear patterned, rhythmic activity corresponding to sighs. Furthermore, calcium transients appeared to occur throughout cell bodies and or processes but appeared as ‘sparks’ of activity rather than coordinated waves. Different types of Ca^2+^ activity in astrocytes could be representative of astrocyte heterogeneity. A heterogeneous glial response, similar to what we observed experimentally, corresponds with what is currently known of astrocyte function in the medulla^49,62,63,71,86,99^. The functional diversity of astrocytes may bear resemblance to the variety of neuronal subtypes found in the medullary respiratory groups. This location-specific sensitivity may reflect astrocyte channel expression (e.g., pH-sensitive TASK channels)^100–102^, connexin/pannexins^103–105^, purinergic receptors^71,73,74,91,92,94,106^ or the type of gliotransmitter released (e.g., d-serine release^63^). In other brain regions, specialized glia cells have been reported for the generation of the circadian rhythm^56,107,108^, where rhythmic occurrences of glia-specific enzymes have been described to alter the circadian rhythm^108–110^.

The heterogeneity of astrocytic subtypes is generally based on their sensitivity to hypoxia or hypercapnia and reflects a compartmentalized view of astrocytic function, where astrocyte function is regionally specific. However, studies have suggested that this response may be more distributed along the ventral surface of the medulla, as is described with D-serine release from astrocytes along the ventral medulla and raphe nucleus in response to CO_2_^63^. It follows that the sigh as an emergent network property is consistent with the idea that the preBötC is organized in a column and can be reconfigured depending on metabolic requirement^32^. Indeed, sigh bursts propagate farther rostral from the preBötC center than eupneic bursts^32^. Determining the extent of different neuronal populations or purportedly sigh-only neurons that perhaps express sigh-specific receptors will enhance our understanding of how neural activity is recruited along the column, and how functional astrocyte heterogeneity may contribute.

The hypothesis that glia contribute to both eupnea and sigh activities is further supported by optogenetic experiments where we show that ChR2 activation of preBӧtC astrocytes results in both eupneic and sigh bursts. This finding has been previously corroborated by others with DREADD upregulation of astrocyte activity, thereby altering the eupneic rhythm^47^. These optogenetic experiments will facilitate further research to determine the specificity of receptors that enhance sigh rhythmogenesis, and whether they reside on neurons or glia. For example, as muscarinic receptors activate the sigh rhythm while inhibiting the eupneic rhythm^30^, one would predict that astrocytes and a subset of ‘sigh-only’ neurons may selectively express muscarinic receptors. In cadmium, we observed that the secondary augmented component of the sigh is absent, along with the extended expiratory duration (‘post-sigh apnea’). The absence of this long expiratory time could be explained by the dynamics of the refractory period^22^, where the recruited population driving synaptic activity is pre-synaptically inhibited by cadmium, and therefore the next burst is able to occur much sooner in the respiratory cycle. However, the augmented phase of the sigh could be largely driven by the recruitment of a larger population of inspiratory neurons through P/Q type Ca^2+^ channel activation, while sigh onset is driven by purinergic mechanisms. This is supported by our optogenetic experiments, where purinergic inhibition blocks the ability to evoke sighs. Furthermore, purinergic block reduces the augmented phase of the in vitro hypoxic response. However, further studies will be necessary to confirm these predictions. In optogenetic experiments, we found a large gradient in the strength of the response to the light stimulus. In 44% of slices, we were able to elicit a robust response (i.e., both eupnea and sighs were evoked with a 200 ms pulse applied contralaterally to the recording electrode). The remaining 55% of Aldh1l1^cre^Ai32 slices had varied responses, which included little to no response, response with an increased light pulse duration, or delayed responses occurring after cessation of the light pulse. We speculate that this variability could be a result of the insertion method used to generate the Aldh1l1^cre^ strain, resulting in varied copy number altering expression strength when crossed with Ai32 transgenic mice. However, optogenetic manipulation of astrocytes in the literature is not thoroughly examined, and thus how membrane depolarization of astrocytes alters activity is not entirely clear. Gourine and colleagues have reported that optogenetic stimulation of astrocytes results in a pH decrease followed by ATP release, which fits well with our hypothesis of sigh generation in the preBötC^51^. A second study showed that optogenetic stimulation of astrocytes in the visual cortex resulted in the facilitation of EPSCs, which was blocked with metabotropic glutamate receptor1a antagonists. This could explain the strong facilitation of sighs by metabotropic glutamate receptor type 8 (mGluR8)^42^.

In conclusion, we propose that the generation of sighs is an emergent property of the preBötC network modulated by neuroglial interactions. These interactions seem to arise from glial, β-adrenergic activated Ca^2+^ transients that are relayed to respiratory neurons through purinergic signaling. The present study provides a conceptual framework that can be further tested experimentally in order to unravel how precise neuro- and glial modulators differentially affect breathing and sighing. It also serves as an example of the role glia play in coordinating the central component of the hypoxic response.

## Methods

### Animals

All experiments and animal procedures were approved by the Seattle Children’s Research Institute’s Animal Care and Use Committee and conducted in accordance with the National Institutes of Health guidelines (OLAW Animal Welfare Assurance number D16-00119). Male and female mice were selected randomly. Experiments were performed in brainstem slices obtained from wild-type CD1 or transgenic mice aged postnatal day 4–12 and bred at Seattle Children’s Research Institute. Aldh1l1^cre^ mice were obtained from Jackson Laboratories (FVB-Tg (Aldh1l1-cre) JD1884Htz/J, Jax stock No. 023748). Cre mice were crossed with homozygous mice containing a floxed STOP channelrhodopsin-2 fused to an EYFP (B6.Cg-Gt(ROSA)26Sor^tm32(CAG-COP4*H134R/EYFP)Hze^/J, Jax stock no. 024109) reporter sequence. All mice were group housed with access to food and water ad libitum in a temperature (22 ± 1 °C) and humidity (40–60%) controlled facility with a 12 h light/dark cycle.

### In vitro transverse brainstem slice preparation and population recordings

The following techniques used are described in detail in a series of publications by the Ramirez laboratory^42,45,111^. PreBötC slice thickness varied between 590–610 μM. Animals were quickly decapitated, and brainstems were rapidly dissected in cold artificial cerebrospinal fluid (ACSF). Serial transverse slices were made from the appearance facial nerve until reaching the level of the preBӧtC, where a 580–610 μM slice was made. Slices were transferred into the recording chamber with circulating ACSF with osmolarity of 305–320 osm/L, pH 7.4 and containing (in mM) NaCl (118), KCl (3), CaCl_2_ (1.5), MgCl_2_ (1), NaHCO_3_ (25), NaH_2_PO_4_ (1), and D-glucose (30). Temperature was maintained at 29–31 °C, and ACSF was bubbled with carbogen (95% O_2_/5% CO_2_). During extracellular population recordings, the superfused ACSF was circulating and recycled at a flow rate of 10–13 ml/min. The activity was recorded from the caudal side of the transverse slice. Some experiments were performed with two slices at the same time using two extracellular electrodes. Extracellular raw traces were filtered and integrated. The data obtained were analyzed using GraphPad Prism 7 and PCLAMP 10 software which is available online at https://www.moleculardevices.com/products/axon-patch-clamp-system/acquisition-and-analysis-software/pclamp-software-suite. For unknown reasons, some preBӧtC slices do not have periodic sighs within the baseline recording period, or they occur at intervals >20 mins. These slices were not used for experiments. PreBötC slices were not used across multiple drug experiments.

### In vivo anesthetized preparation

In vivo anesthetized experiments were performed as previously published^22^. Briefly, adult mice were anesthetized with urethane (1.5 mg/kg, i.p.) and placed supine on a custom surgical table. Adequate depth of anesthesia was determined via heart rate, breathing frequency, and response to toe pinch. Bipolar stainless steel electrodes were implanted in the diaphragm muscles to perform electromyographic recordings of inspiratory motor activities^112^. The trachea was exposed through a midline incision and cannulated caudal to the larynx with a curved (180°) tracheal tube (24G). The trachea and esophagus were removed rostral to the tracheotomy, and underlying muscles were removed to expose the basal surface of the occipital bone. Mice were supplied with 100% O_2_ throughout the remainder of the surgery and experimental protocol. The portion of the occipital bone and dura overlying the ventral medullary surface were removed, and the brainstem surface was perfused with aCSF (36 °C) equilibrated with carbogen (95% O_2_, 5% CO_2_). The hypoglossal nerve (XII) was then isolated unilaterally and recorded using a fire-polished pulled glass pipette filled with aCSF. XII electrical activity was amplified (10,000×), filtered (low pass 300 Hz, high pass 5 kHz), rectified, integrated, and digitized (Digidata 1550A, Axon Instruments). In animals in which we observed baseline sighs, MRS2279 (500 μM, 41.3nL) and Evan’s Blue dye or red fluorospheres were co-injected bilaterally in three positions around the preBötC using a NanoInject, as to avoid lesioning the preBötC core and altering the rhythm. In most instances, injections close to preBötC stimulated sighs immediately following placement of the cannula. Saline-injected control animals did not have any alterations in baseline breathing, and we observed the same increase in sighs in most injections (*n* = 4/5). Similar to in vitro experiments, many animals in vivo did not have baseline sighs, and therefore MRS2279 was not injected.

### Optogenetics and pharmacology

A glass fiber optic (200 μm core, 0.24NA) connected to a blue (447 nm) high-powered laser was positioned above the preBötC contralateral to the extracellular electrode or XII nerve and positioned for maximal response. Power was set to 0.5 mW/mm^2^. Fifty continuous 20 s sweeps were recorded for each stimulation experiment in vitro, and 20 sweeps for in vivo experiments. Power settings and pulse width were selected based on previous experiments^11,32^. In approximately 1/3 of Aldh1l1^cre^/Ai32 preBötC slices, we had difficulty evoking any responses unless increasing the wavelength to a higher laser power (>0.5 mW/mm^2-^ 1 mW/mm^2^). Despite having robust respiratory rhythms, we did not continue to perform stimulations in these slices. Control stimulations were performed in Aldh1l1^cre-/-^/Ai32 littermates for in vitro (*n* = 2) and adults for in vivo (*n* = 3) experiments. There were no observed non-specific effects of optogenetic stimulation, even at maximum power.

Norepinephrine (1–40 μM, DL-Norepinephrine hydrochloride, cat. A7256), Isoproterenol (Isoprenaline hydrochloride, cat. I5627), Strychnine (1 μM, cat. S0532), and cadmium (4 μM cadmium chloride, cat. 20208) were purchased from Sigma. Gabazine (1 μM, SR 99531 hydrobromide, cat. 1262), MRS2279 (20 μM, P2Y_1_R antagonist, cat. 2158,), MRS2365 (20 μM, P2Y_1_R agonist, cat. 2157) and TNP-ATP triethylammonium salt (20 μM, P2X receptor antagonist, cat. 2464) were obtained from Tocris. All drugs were diluted in water.

### Calcium imaging

All Ca^2+^ imaging was performed in p4-p13 postnatal pups from transgenic mice (Aldh1l1^cre^/R26^Lck^, Aldh1l1^cre^/TIGRE^Ai162^) in the transverse preBötC slice preparation as above. Imaging was performed on a Bruker Ultima Investigator two-photon microscope with a 20x NA = 1.0 objective (Olympus XLUMPlanFL N) and a 920 nm laser (MaiTai DeepSee HP 10040 S Spectra-Physics). Slices were adhered to raised plastic grid and continuously perfused with aCSF bubbled with carbogen as described above. Large bore glass electrodes were used to record population activity from the preBötC. Then, 512 × 512-pixel images were collected in galvanometer control mode (0.8 frames per second, *n* = 6; 7.4 frames per second, *n* = 1) or resonance galvanometer control mode (29.7 frames per second, *n* = 2). Images were collected such that the lateral ventral edge of the slice was just visible in the corner of the field of view (~580 × 580-micron FOV). Calcium imaging data was collected using Prairie View vs. 5.5. Images were motion corrected with Suite2P (vs. 0.12.0)^113^. For images collected in galvanometer mode, ROIs were hand drawn and extracted in ImageJ; for images collected in resonance galvanometer mode (29.7 fps), ROIs were automatically detected by Suite2P. All fluorescence signals were extracted and neuropil corrected by Suite2p. We then processed all neuropil-corrected fluorescence signals as z-scores: ${F}_{z}=\frac{F-\bar{F}}{{\sigma }_{F}}$ and performed all subsequent analyses using the z-scored fluorescence signal. For signals acquired in resonance galvanometer mode (29.7 fps), we performed a 10-sample centered moving average smoothing. To determine the change in astrocyte Ca^2+^ activity elicited by bath application of 20 μM isoproterenol, we computed the standard deviation of each preprocessed Ca^2+^ signal in a 5-min time window at the beginning of a recording (baseline), and a 5-min time window immediately after addition of isoproterenol to the bath. We computed the standard deviation of the trace in those time windows and computed the percent difference between these standard deviations as: $100\cdot \frac{{{\sigma }_{{isoproteronol}}-\sigma }_{{baseline}}}{{\sigma }_{{baseline}}}$. Rhythmic ROIs were detected by first performing an autocorrelation on the z-scored Ca^2+^ fluorescence trace (python *statsmodels* package) and computing the 95% confidence interval (CI) for that autocorrelation. We then considered an ROI rhythmic if a peak in the autocorrelation is detected such that the upper bound of the autocorrelation CI first drops below zero, followed by the lower bound of the CI crossing above zero. If this occurred, the ROI was considered rhythmic, and the time lag and strength of that autocorrelation were extracted.

### Histology

After experiments, animals were fixed by transcardial perfusion with cold ACSF followed by 4% PFA in phosphate buffer solution (PBS). Medullary and cortical tissue was carefully extracted and equilibrated in graded sucrose solutions to a final concentration of 30%, embedded in OCT solution, frozen at −80 °C, and cryosectioned at 25 μm for imaging of immunofluorescence. Tissue processed in this way was suitable for imaging of immunofluorescence. Brains used in fluorescence in situ hybridization (FISH) experiments were freshly dissected, flash frozen in isopentane, mounted with OCT solution, and cryosectioned at 25 μm. Slides containing brainstem tissue slices were washed 3 × 10 min in PBS. Tissue slices were then permeabilized and blocked with 1% normal donkey serum in 0.5% Triton X-100/PBS for 1 h. Following blocking, tissue slices were incubated with the primary antibody for either NeuN (clone A60, Millipore MAB377, 1:1000), BIII Tubulin (Abcam ab18207, 1:1000), Aldh1l1 (ab87117, 1:500), or P2Y1 (Abcam ab140859, 1:500) in 1% normal donkey serum in 0.5% Triton X-100/PBS overnight. After primary antibody incubation, tissue was washed 3 × 10 min in phosphate buffer solution (PBS), followed by secondary antibody incubation for 30 min (Alexa Fluor 488, Invitrogen a21206, donkey anti-rabbit IgG (H+L), Alexa Fluor 594, Invitrogen a21203 donkey anti-mouse IgG (H+L), or Alexa Fluor 647, Molecular Probes a31573 donkey anti-rabbit IgG (H+L), all 1:500 dilution). Lastly, tissue was washed 3 × 10 min in PBS and hard-set mounted (VECTASHIELD hardset mounting media with DAPI). Immunofluorescence images were acquired using an Olympus VS120 Virtual Slide Microscope Scanner. The excitation/emission filter sets used for imaging were DAPI: 380-405/410-480, FITC: 460-490/500-550, Cy3/TRITC: 540-570/580-640, and Cy5: 625-645/655-705. Laser scanning confocal imaging was performed using a Zeiss 710 34-channel Quasar LSC Microscope. FISH was performed with the RNAscope Multiplex Fluorescent V2 kit, according to the manufacturer’s instructions (Advanced Cell Diagnostics). The mouse probes used included: Aldh1l1 (catalog #405891), Rbfox3 (catalog #313311-C2), P2ry1 (catalog #406061-C3) and were used at 1:50.

### Sigh classification

Large amplitude eupneic bursts can be mistaken for sigh bursts, in particular in slices that show irregular rhythmicity with variable levels of synchrony among respiratory neurons and variable amplitude of eupneic activity, or in slices that show burstlet activity intermixed with synchronized eupneic bursts. Thus, we only characterized slices that showed regular well-synchronized eupneic and sigh activity to avoid any ambiguity. Moreover, custom unsupervised methods were developed to distinguish between eupneic bursts and “sigh attempts” evoked by channelrhodopsin stimulation in in vitro recordings. First, bursts in the integrated preBötC recording were detected such that the prominence of each peak was greater than 2.5 times the standard deviation of the integrated activity. The onset of the burst was defined as the time at which the trace reached 20% of the subsequent peak height. Bursts that were aberrantly large amplitude (~>75 times the mean amplitude) or very fast rising (reached peak amplitude in less than 150 ms) were discarded as noise. The burst shape for all bursts was extracted from the onset to 1.5 s after the onset of the burst. Burst shapes were then decomposed into principal components. The first *n* components (where *n* is the number of components needed to explain 95% of the variance) of the spontaneous bursts (i.e., not evoked by optogenetic stimulation) were used as features to fit a robust covariance estimator (sci-kit learn MinCovDet). Since the spontaneous bursts were never putative sighs, this covariance represents the prior distribution of eupnea, that is, the expected eupneic shape. We calculate the Mahalnobis distance (*D*) of each burst to the mean eupneic shape. *D* quantifies how similar each burst is to the “average” eupnea. We then determine a threshold as:

1 $${D}_{{thresh}}=\widetilde{{D}_{{spontaneous}}}+4 * {IQR}\left({D}_{{spontaneous}}\right)$$

All evoked bursts where ${D}_{{evoked}} > {D}_{{thresh}}$ were considered outliers. This outlier detection included many false positive bursts in which the burst shape was different from the average eupnea, but upon inspection, it was not typical of a sigh (often long slow bursts or large amplitude noise). We boost this outlier detection with an identical method using instead the intuitive features of burst amplitude, full width at half max (FWHM) and time to next burst (post-burst apnea) as features to the covariance estimator. A burst was considered a sigh attempt when it was determined to be an outlier in both the classifier fit on burst shape and on the intuitive features. We imposed a set of final criteria where a sigh attempt must be larger in amplitude than 90% of the spontaneous bursts. To classify sighs in in vivo recordings, diaphragm EMG was recorded, rectified, and integrated as described above. Bursts in the integrated diaphragm EMG were detected, and each burst shape was extracted. The area under the curve was computed for each burst. Bursts were labeled as “sighs” if both the area under the curve and the amplitude for a given sigh were >6x the median absolute deviation (MAD) of the 20 bursts preceding and following the given burst.

### Statistical analysis

Analyses were performed using GraphPad Prism 9 and custom Python software. Groups were compared using paired two-tailed *t*-tests or ordinary one-way or ANOVAs followed by Bonferroni’s post hoc tests, if necessary. Statistical significance was set at α = 0.05. Data are presented as means ± SE. Masking was not used during data collection or analysis.

### Caveats and limitations

The use of transverse preBötC slices as a model for respiratory rhythm generation is associated with significant caveats of which oxygenation^114^, incomplete network, low temperature, elevated [K+] saline are the most obvious concerns, and have been discussed in most of our publications^29,45,66,114,115^. Although transverse slices generate rhythmicity in 3 mM K^+^^37,116,117^, rhythmicity is more reliable and consistently generated at 8 mM K^+^. Thus, in the present study, we consistently use the extracellular concentration of 8 mM K^+^. To distinguish the fast-respiratory rhythm from the slow sigh rhythm, we refer to the fast-respiratory rhythm as “eupneic” activity throughout this study. However, we do not imply that this in vitro activity represents “eupnea” in the behavioral sense since slices are not connected to respiratory motor output. Rather this activity characterizes primarily the inspiratory component of the respiratory activity generated within the preBötC^25,29,118^. Furthermore, sighs were characterized based on their appearance in the in vivo preparation and were carefully distinguished using a custom pipeline aimed at reducing bias. However, in vivo recordings should involve increased diaphragm activity along with the characteristics observed in sighs, and thus in vitro work is limited in this sense. In our analysis, we did not consider doublets of eupneic activity to be sighs.

### Reporting summary

Further information on research design is available in the Nature Portfolio Reporting Summary linked to this article.

### Supplementary information

Supplementary Information

Reporting Summary

### Source data

Source Data

## Data Availability

The raw data presented in all figures of this manuscript are available upon request. Source data are provided with this paper.

## Code availability

The source code presented in all figures of this manuscript is available upon request.
