# Supplementary material for: Purinergic signaling mediates neuroglial interactions to modulate sighs
Source: Nat Commun. 2023 Aug 31;14:5300. doi: 10.1038/s41467-023-40812-x (PMC10471608; doi:10.1038/s41467-023-40812-x)
Supplement: Supplementary file 1 — Supplementary Information [file 41467_2023_40812_MOESM1_ESM.pdf]

Purinergic signaling mediates neuroglial interactions to modulate sighs

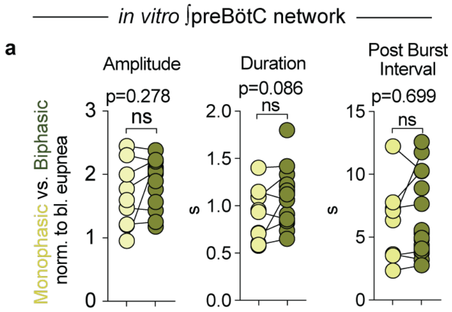

Supplemental Figure 1. **Characterization of spontaneous monophasic versus biphasic sighs. a** Amplitude, duration, and post burst intervals of monophasic vs biphasic sighs are not significantly different (n=18, paired t-test).

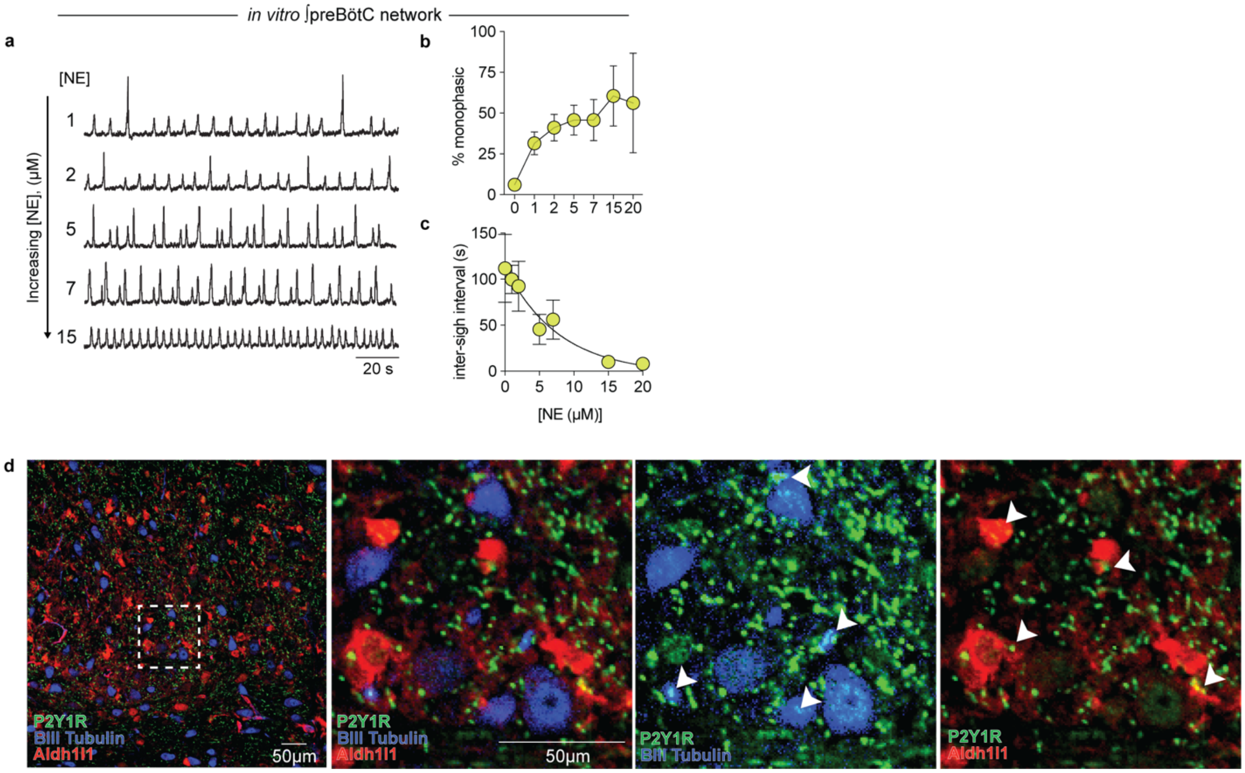

Supplemental Figure 2. **Norepinephrine (NE) modulates sigh and eupneic frequency. a** increasing concentrations of NE increase sigh frequency. At higher concentrations, NE disrupts eupneic frequency and eliminates sighing (n=9). **b** Percent of monophasic sighs increases with increasing NE concentrations. **c** Inter-sigh interval decreases with increasing NE concentration up to 20 μM NE with cessation of sighs. **d** PreBötC slice immunostained with βIII Tubulin (neurons) and Aldh1l1 (astrocytes), showing observed colocalization with P2Y1R. Right panels are zoom of inset with separated channels. White arrows point to examples of P2Y1R signal colocalization with cytoplasmic staining of either βIII Tubulin or Aldh1l1. Data are presented as mean values +/- SEM.

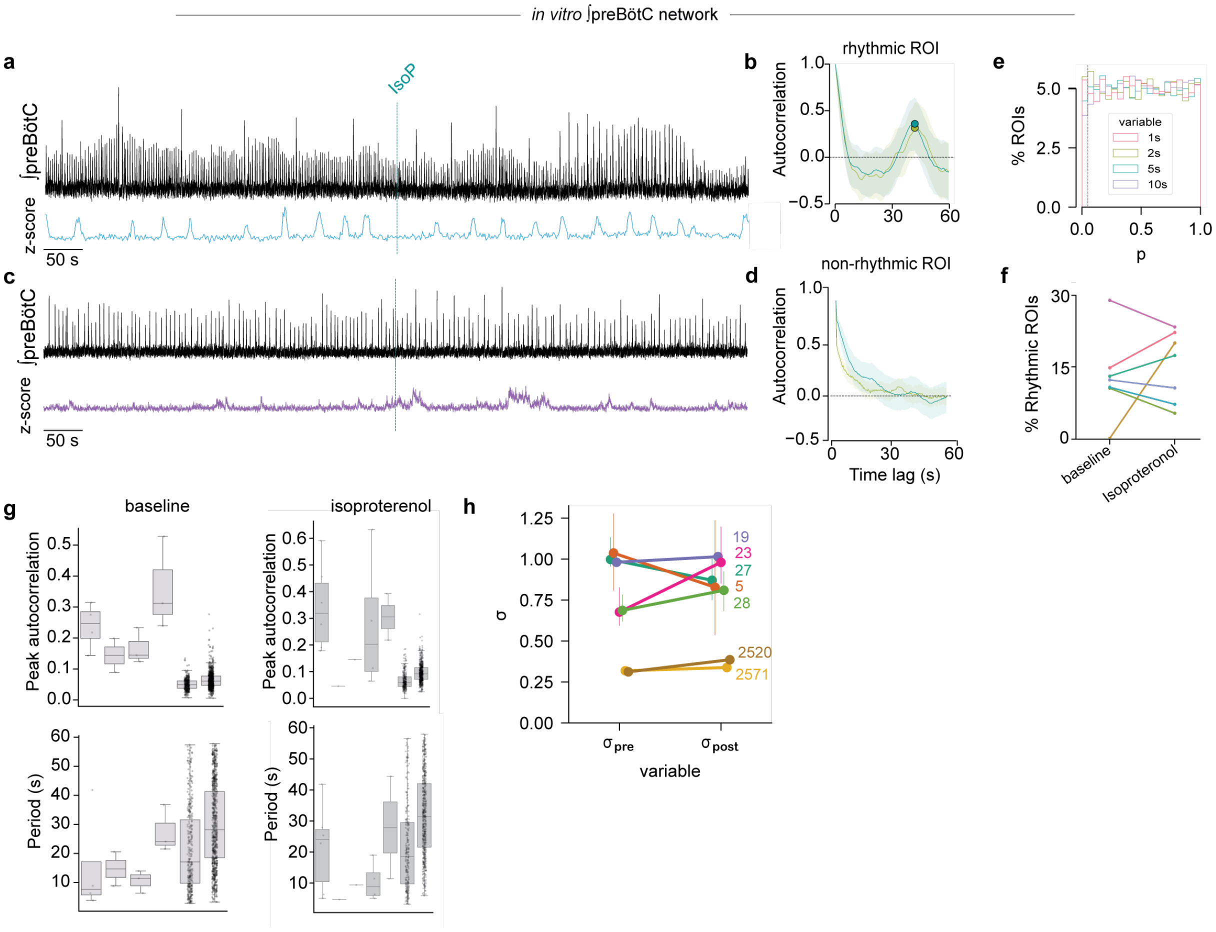

Supplemental Figure 3. **Astrocytic Ca^{2+} signals are periodic but not temporally correlated to sighs. a** Integrated preBötC activity with regular sigh events (top) and a simultaneously recorded periodic Ca^{2+} signal from one ROI **b** Autocorrelation of the Ca^{2+} signal exhibits a strong peak indicating rhythmic activity with a period of ~45s. Green is baseline, turquoise is in isoproterenol. Shaded region is +/- 95% confidence interval. **c,d** as in (a,b), but for an ROI that does not exhibit periodicity. **e** Distribution of two-tailed paired t-test p-values for all ROI Ca^{2+} signals before and after sigh occurrence, for windows of 1,2,5 and 10 seconds. ROIs are not significantly modulated by the occurrence of sighs, as indicated by a uniform distribution of p-values. Color indicates window length used to compute paired t-test **f** % of total ROIs that were determined to be periodic in baseline and isoproterenol for each slice; individual slices

are colored differently. **g** (top)The peak correlation strength, and (bottom) fundamental period, for each periodic ROI, separated by slice, in baseline (left) and isoproterenol (right). Box is median +/- IQR, whisker is +/- 1.5*IQR (Baseline ROI's =4, 2, 3, 3, 315, 730; isoproterenol ROI's+6, 1, 1, 4, 2, 272, 589). **h** Ca^{2+} activity (as standard deviation) before (pre) and after (post) addition of isoproterenol for each slice. Markers are means of all ROIs in a given slice, error bars are 95% confidence interval. N
umber of ROI's labeled to the right of graph.

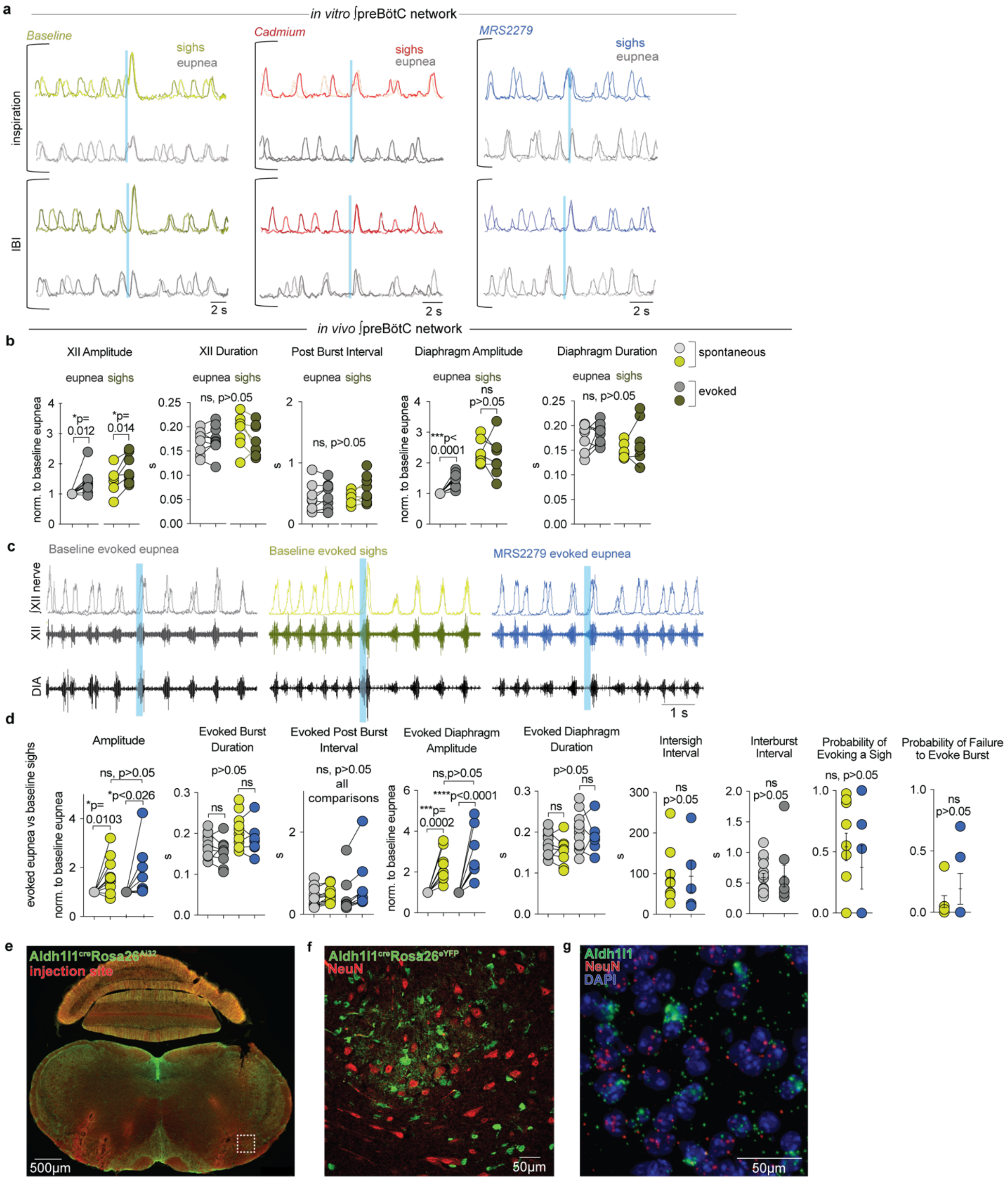

Supplemental Figure 4. **Chr2 activation of preBötC astrocytes drives sighing *in vitro* and *in vivo*. a** Representative population recording from the preBötC *in vitro* showing eupnea (gray and black) and sighs/sigh attempts generated during inspiration and during the inter-burst interval (IBI). Blue line represents 200ms laser pulse. **b**

Quantification of in vivo spontaneous vs. evoked eupnea and sighs (n=11, paired t-test). Diaphragm amplitude was most defining characteristic of sighs *in vivo*, as in XII nerve recordings evoked eupneic bursts had larger amplitudes than spontaneous eupneic bursts. **c** Evoked eupnea and sighs *in vivo* under baseline conditions and with application of MRS2279 (n=11, paired t-test). MRS2279 was ineffective at blocking most sighs and limited the spontaneous and evoked response in a small number of experiments, regardless of injection location. (d) Quantification of baseline and evoked eupnea and sighs *in vivo*. (e) Representative MRS2279 injection sites (red) in Aldh1l1^{cre}Rosa26^{Ai32} (green), white box indicates relative location in (f, g). (f) Immunostaining with NeuN in Aldh1l1^{cre}/Rosa26^{eYFP} preBötC slice to demonstrate specificity of Aldh1l1^{cre} transgenic line. Nuclei are labeled in blue (DAPI). (g) preBötC slice with FISH demonstrating further specificity of Aldh1l1 astrocytic marker. Approximate location of right insets shown in dotted white box. Right panels show inset of preBötC region taken with laser scanning confocal microscopy (40x). Data are presented as mean values +/- SEM.
