# Supplementary material for: Purinergic signaling mediates neuroglial interactions to modulate sighs
Source: Nat Commun. 2023 Aug 31;14:5300. doi: 10.1038/s41467-023-40812-x (PMC10471608; doi:10.1038/s41467-023-40812-x)
Supplement: Supplementary file 2 — Reporting Summary [file 41467_2023_40812_MOESM2_ESM.pdf]

nature portfolio

Corresponding author(s): Jan Marino Ramirez

Last updated by author(s): Aug 8, 2023

Reporting Summary

Nature Portfolio wishes to improve the reproducibility of the work that we publish. This form provides structure for consistency and transparency in reporting. For further information on Nature Portfolio policies, see our Editorial Policies and the Editorial Policy Checklist.

Statistics

For all statistical analyses, confirm that the following items are present in the figure legend, table legend, main text, or Methods section.

n/a	Confirmed	
	x	The exact sample size (n) for each experimental group/condition, given as a discrete number and unit of measurement
	x	A statement on whether measurements were taken from distinct samples or whether the same sample was measured repeatedly
	x	The statistical test(s) used AND whether they are one- or two-sided<br>*Only common tests should be described solely by name; describe more complex techniques in the Methods section.*
	x	A description of all covariates tested
	x	A description of any assumptions or corrections, such as tests of normality and adjustment for multiple comparisons
	x	A full description of the statistical parameters including central tendency (e.g. means) or other basic estimates (e.g. regression coefficient) AND variation (e.g. standard deviation) or associated estimates of uncertainty (e.g. confidence intervals)
	x	For null hypothesis testing, the test statistic (e.g. F, t, r) with confidence intervals, effect sizes, degrees of freedom and P value noted<br>*Give P values as exact values whenever suitable.*
	x	For Bayesian analysis, information on the choice of priors and Markov chain Monte Carlo settings
	x	For hierarchical and complex designs, identification of the appropriate level for tests and full reporting of outcomes
x		Estimates of effect sizes (e.g. Cohen's d, Pearson's r), indicating how they were calculated

Our web collection on statistics for biologists contains articles on many of the points above.

Software and code

Policy information about availability of computer code

Data collection	Electrophysiology data was recorded with Clampex10.7. Two-photon calcium imaging was recorded with Prairie View 5.5
Data analysis	Electrophysiology was analyzed with Clampfit 10.7, custom python 3 code, and Matlab 2019 code. Two photon data were analyzed with ImageJ 1.53q abd custom python code that incorporated the Suite2P package. Open source packages for python and Matlab are used throughout, and are indicated or cited in the manuscript. All analysis code is available upon request.

For manuscripts utilizing custom algorithms or software that are central to the research but not yet described in published literature, software must be made available to editors and reviewers. We strongly encourage code deposition in a community repository (e.g. GitHub). See the Nature Portfolio guidelines for submitting code & software for further information.

Data

Policy information about availability of data

All manuscripts must include a data availability statement. This statement should provide the following information, where applicable:

- Accession codes, unique identifiers, or web links for publicly available datasets
- A description of any restrictions on data availability
- For clinical datasets or third party data, please ensure that the statement adheres to our policy

The raw data and source code presented in all figures of this manuscript are available upon request.

Human research participants

Policy information about studies involving human research participants and Sex and Gender in Research.

Reporting on sex and gender	N/A
Population characteristics	N/A
Recruitment	N/A
Ethics oversight	N/A

Note that full information on the approval of the study protocol must also be provided in the manuscript.

Field-specific reporting

Please select the one below that is the best fit for your research. If you are not sure, read the appropriate sections before making your selection.

☒ Life sciences ☐ Behavioural & social sciences ☐ Ecological, evolutionary & environmental sciences

For a reference copy of the document with all sections, see nature.com/documents/nr-reporting-summary-flat.pdf

Life sciences study design

All studies must disclose on these points even when the disclosure is negative.

Sample size	We selected samples sizes based on power analysis and 30+ years of experience with slice and in vivo electrophysiology/physiolgy experimentation.
Data exclusions	preBötC slices that did not have sighs at baseline or had disrupted rhythms (e.g. sighs could not be distinguished from eupnea using our established criteria) were not used in our study for additional drug treatments/manipulation.
Replication	Almost all experiments were performed by more than one experimenter, all attempts at replication were successful. The Ramirez lab holds an expertise for all techniques performed in this study. To ensure reproducibility, all experimenters are rigorously trained by most senior experimenter in the lab. Furthermore, to ensure reproducibility, multiple experimenters confirmed almost all findings in the article. Data that could not be replicated by at least two investigators were excluded. In vitro slices and in vivo experiments were performed in multiple electrophysiology rigs to control for variability in flow rates, oxygenation, etc..
Randomization	Animals were randomly selected from mouse litters and were not selected for based on sex or other attributes. No group allocation was necessary for experimental data collection.
Blinding	Data analysis with supplied code (all in vivo optogenetics and in vivo data) was blinded to the experimenter performing the analysis. Slice data analysis of drug conditions was not blinded for the following reasons: (1) blinding was not necessary, as an automated analysis of sigh and eupnea traces was performed using thresholding in clampfit, which was kept consistent in analysis of drug treatments to prevent experimenter bias. Clampfit software is automatically 'triggered' for each respiratory burst. 'Trigger' levels (amplitude that a burst needs to reach to be considered a burst) must be set by experimenter prior to the analysis and is fully automated by the software, and regularly checked by the investigator to control for malfunctioning. (2) Blinding of analysis would be difficult/potentially not possible because each file contained tags for time of each drug addition that are not removable after data collection. In order to blind experimenter to analysis groups, code would have had been written to extract the labels (if this was possible at all) from clampfit software and at the time of data collection the Ramirez lab did not have lab members capable of coding. Animals were randomly selected and allocated to an experimental group. But, the selection depended in large part on the availability of mice that had to have the right age range and genotype required for the study. Because they were bred and crossed at the institute and supervised by veterinary staff, it was not always predictable when mice became available - for some of the genotypes we had to wait several months to become available for an experiment. Thus, experimenters did not need to be blinded for group allocation as allocation was mostly dictated by availability.

Reporting for specific materials, systems and methods

We require information from authors about some types of materials, experimental systems and methods used in many studies. Here, indicate whether each material, system or method listed is relevant to your study. If you are not sure if a list item applies to your research, read the appropriate section before selecting a response.

Materials & experimental systems

n/a	Involved in the study	
	☒	Antibodies
☒		Eukaryotic cell lines
☒		Palaeontology and archaeology
	☒	Animals and other organisms
☒		Clinical data
☒		Dual use research of concern

Methods

n/a	Involved in the study	
☒		ChIP-seq
☒		Flow cytometry
☒		MRI-based neuroimaging

Antibodies

Antibodies used

NeuN (Millipore MAB377, A60), P2Yl (Abeam ab140859), Aldh1l1 (ab87117), , BIII tubulin (Abcam ab18207)
RNAscope probes from Advanced Cell Diagnostics: Aldhlll (catalog #405891), Rbfox3 (catalog #313311-C2), P2ryl (catalog #406061-C3). secondaries: (Alexa Fluor
488, Invitrogen a21206, donkey anti-rabbit IgG (H+L), Alexa Fluor 594, Invitrogen a21203 donkey anti-mouse IgG (H+L), or Alexa Fluor 647, Molecular Probes
a31573 donkey anti-rabbit IgG (H+L), all 1:500 dilution).

Validation

Secondary only controls were performed with each iteration of staining to identify any non-specific binding, and tested with positive control tissues if suggested on datasheet.
NeuN, clone A60 (Millipore MAB377, A60): https://www.emdmillipore.com/US/en/product/Anti-NeuN-Antibody-clone-A60,MM_NF-MAB377#anchor_REF
BIII tubulin, rabbit polyclonal to beta III tubulin (Abcam ab18207): https://www.abcam.com/products/primary-antibodies/beta-iii-tubulin-antibody-neuronal-markerab18207.html
P2Yl (Abcam ab140859) rabbit polyclonal to P2Y1: https://www .abcam.com/products/pri ma ry-a ntibodies/p2yl-antibody-c-terminal-a b140859.html
Aldh1l1 (ab87117) rabbit polyclonal to Aldh1l1: https://www.abcam.com/products/primary-antibodies/aldh1l1-antibody-astrocyte-marker-ab87117.html
RNAscope probes: Aldhlll (catalog #405891), Rbfox3 (catalog #313311-C2), P2ryl (catalog #406061-C3)
Aldh1l1: https://acdbio.com/search/site/%252A405891 %252A/cms/probes
RBfox3: https://acdbio.com/search/site/%252A313311-C2%252A/cms/probes
P2Ryl: https://acdbio.com/search/site/%252A406061-C3%252A/cms/probes

Animals and other research organisms

Policy information about studies involving animals; ARRIVE guidelines recommended for reporting animal research, and Sex and Gender in Research

Laboratory animals

Mus Musculus; Aldhlllcre (FVB-Tg (Aldhlll-cre) JD1884Htz/J, Jax stock No. 023748), Ai32 (Jax stock No. 012569), CDl. in vitro slice preparations were performed with animals aged postnatal d4-12; in vivo anesthetized preparation experiments were performed with animals postnatal day 30 and less than 1 year of age.

Wild animals

no wild animals were used in this study.

Reporting on sex

Male and female mice were selected for randomly. We did not explore sex-specific affects of sigh generation in our article; as some sample sizes would be too small to explore sex-specific effects in this dataset.

Field-collected samples

no field collected samples were used in this study.

Ethics oversight

All experiments and animal procedures were approved by the Seattle Children's Research Institute's Animal Care and Use Committee and conducted in accordance with the National Institutes of Health guidelines.

Note that full information on the approval of the study protocol must also be provided in the manuscript.
